# A Loss-of-Function Variant Causing Primary Autosomal Recessive Hypertrophic Osteoarthropathy

**DOI:** 10.7759/cureus.88312

**Published:** 2025-07-19

**Authors:** Devanshi N Patel, Richard Sidlow

**Affiliations:** 1 Department of Pediatric Genetics, University of Missouri School of Medicine, Columbia, USA

**Keywords:** case report pediatric, genetic syndromes, orthopedic, pediatric, pediatric case, pediatric genetics, primary hypertrophic osteoarthropathy

## Abstract

Primary autosomal recessive hypertrophic osteoarthropathy (PHOAR) type 1 is caused by the failure of the *HPGD* gene product to break down prostaglandins. We report the case of a two-year-old male patient diagnosed with PHOAR1 due to a previously unreported homozygous intragenic deletion. Upon retrospective review of the patient's history, his clinical course proved to be typical of this syndrome, although it had been unrecognized. This case report presents an opportunity to review the natural history, clinical signs, diagnosis, and treatment of PHOAR1, with the aim of raising awareness of this rare entity.

## Introduction

Primary autosomal recessive hypertrophic osteoarthropathy (PHOAR) is a genetic condition characterized by digital clubbing, arthropathy, and periosteal bone proliferation [1]. There are two clinically distinct forms: PHOAR1, whose peaks of presentation occur in the first year of life and adolescence, and PHOAR2, which typically presents in adolescence or later [1,2]. PHOAR1 is caused by pathogenic homozygous/combined heterozygous variants in the *HPGD* gene located on chromosome 4q34, the gene product being the enzyme 15-hydroxyprostaglandin dehydrogenase that helps break down prostaglandins [3]. PHOAR2 is caused by pathogenic variants in *SLCO2A1*, which codes for a prostaglandin transporter protein located in various tissues [1].

For PHOAR1, there is an approximate 1:1 male-to-female ratio, whereas PHOAR2 is seen mostly in males [4]. Symptoms of PHOAR1 include delayed closure of fontanels, congenital heart disease (i.e., delayed/nonclosure of patent ductus arteriosus (PDA)), pachydermia, swelling of the hands and feet, and expanded diaphysis of bones [1,5]. These symptoms appear to persist through age four, after which digital clubbing and knee joint swelling become more permanent, along with acro-osteolysis and hyperkeratosis of the palms, while cranial ossification defects resolve [1,5].

Herein, we describe a case of PHOAR1 diagnosed early in life with a description of his clinical course to date.

## Case presentation

The patient is a two-year-old male born to a 21-year-old G1P0 mother via normal spontaneous vaginal delivery at term. No infectious risk factors, drug use, or exposures were documented during pregnancy, and both delivery and family history were noncontributory.

The patient initially presented at five weeks of age with an *E. coli* urinary tract infection in the context of mild left ureteral dilation and was subsequently found to be in congestive heart failure. An echocardiogram revealed a large PDA with left-to-right shunting, which was subsequently plugged via a transcatheter Amplatzer plug. The physical examination revealed enlarged fontanelles and widely spaced nipples. Growth parameters were weight at the 3rd percentile (Z = -1.86), length at the 9th percentile (Z = -1.35), and head circumference at the 0.07th percentile (Z = -3.19). A chromosomal microarray analysis was performed, revealing multiple regions of homozygosity that totaled 4.83% of the total genome, without any copy number changes of significance. The paternal grandmother and maternal grandmother were sisters, and they are the probable source of the homozygous variant. Echocardiographic resolution of the PDA was documented at 13 months of age with residual mild impingement of the left pulmonary artery.

**Figure 1:**
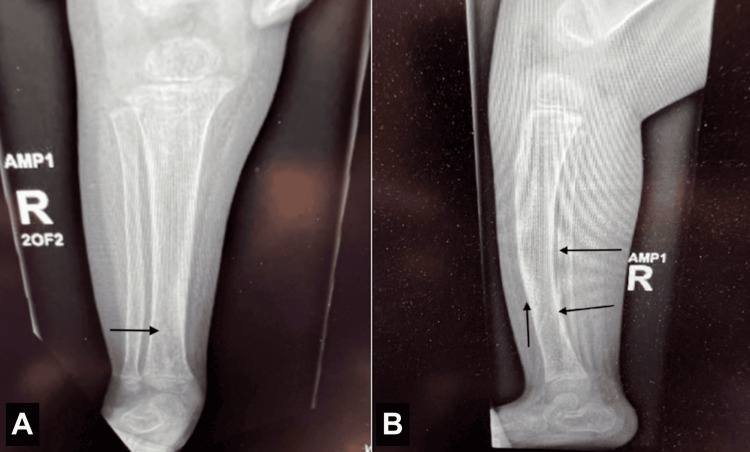
X-ray of the right tibia (A) axial and (B) sagittal views. Arrows refer to a nondisplaced distal tibial toddler's fracture along with mild osteopenia, mild bowing, and diaphyseal thickening.

**Figure 2:**
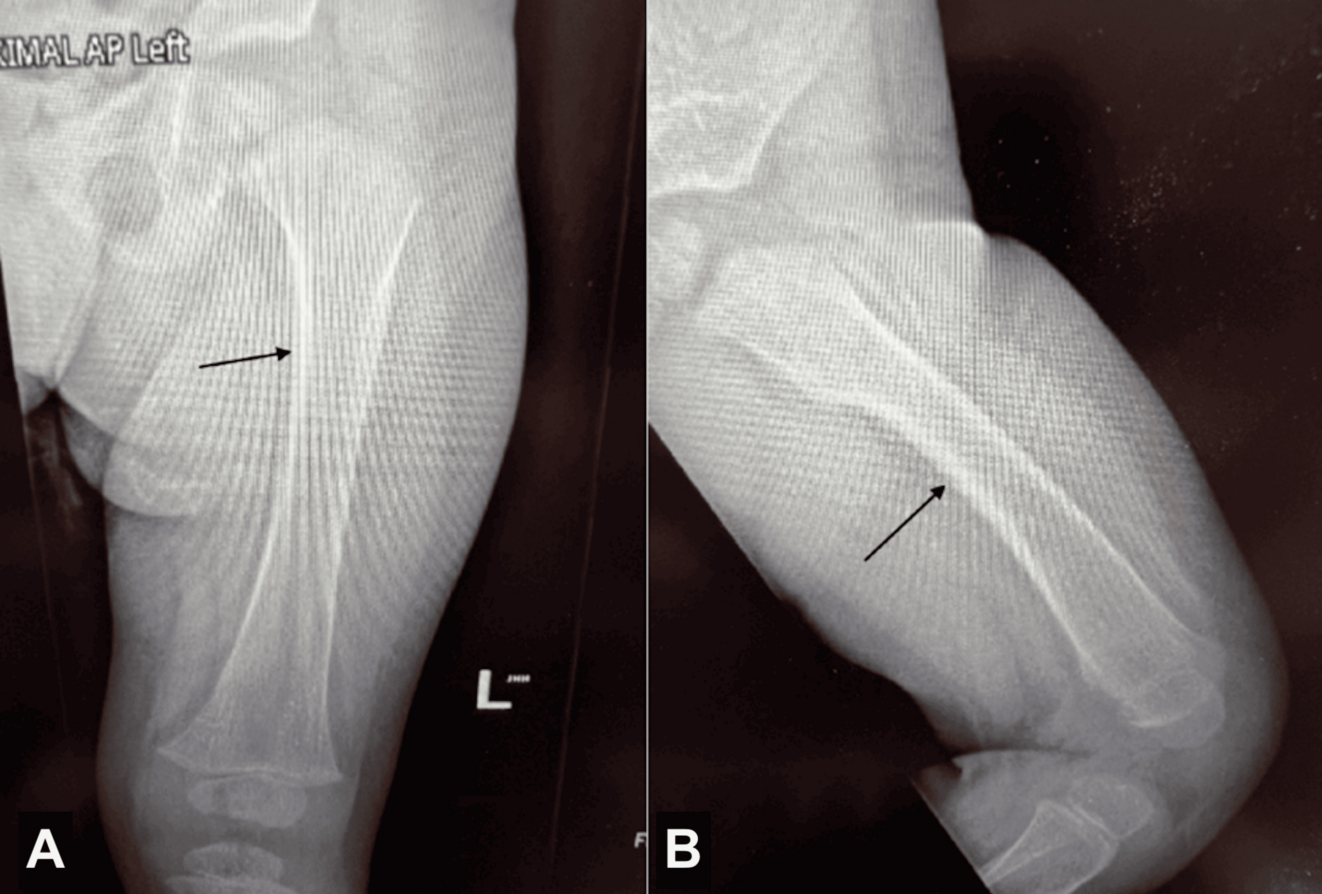
X-rays of the left femur (A) axial and (B) sagittal views. Arrows indicate mild osteopenia, mild bowing, and diaphyseal thickening.

Whole-exome sequencing (WES) was ordered at four months of age. He was seen by his pediatrician at 17 months of age as a follow-up for a right distal tibial toddler's fracture, which was diagnosed the month prior. Mild osteopenia, mild bowing, and diaphyseal thickening of the right lower extremity were noted on X-ray, which prompted concern for metabolic bone disease (Figure 1). These findings were also seen in the X-ray of the left lower extremity (Figure 2). A physical exam at this time revealed an eczematous rash and digital clubbing.

At 18 months of age, the results of WES were reviewed. Positive findings on physical examination at this time included 10-finger and 10-toe digital clubbing, hyperhidrosis, and an inability to fully extend the hips and knees, resulting in a crouched stance and walking posture. Fontanelles were closed.

WES revealed homozygous deletions of exons 5-7 in *HPGD* [GRCh37][chr4:175413105_175416776][NM_000860.5](GeneDx: Gathersburg, MD) [6]. This variant, according to the Database of Genomic Variants, has not been observed at a significant frequency in large population cohorts. This variant has also not been previously published as pathogenic or benign, with loss of function not a well-established mechanism of disease, making it a variant of unknown significance.

**Figure 3:**
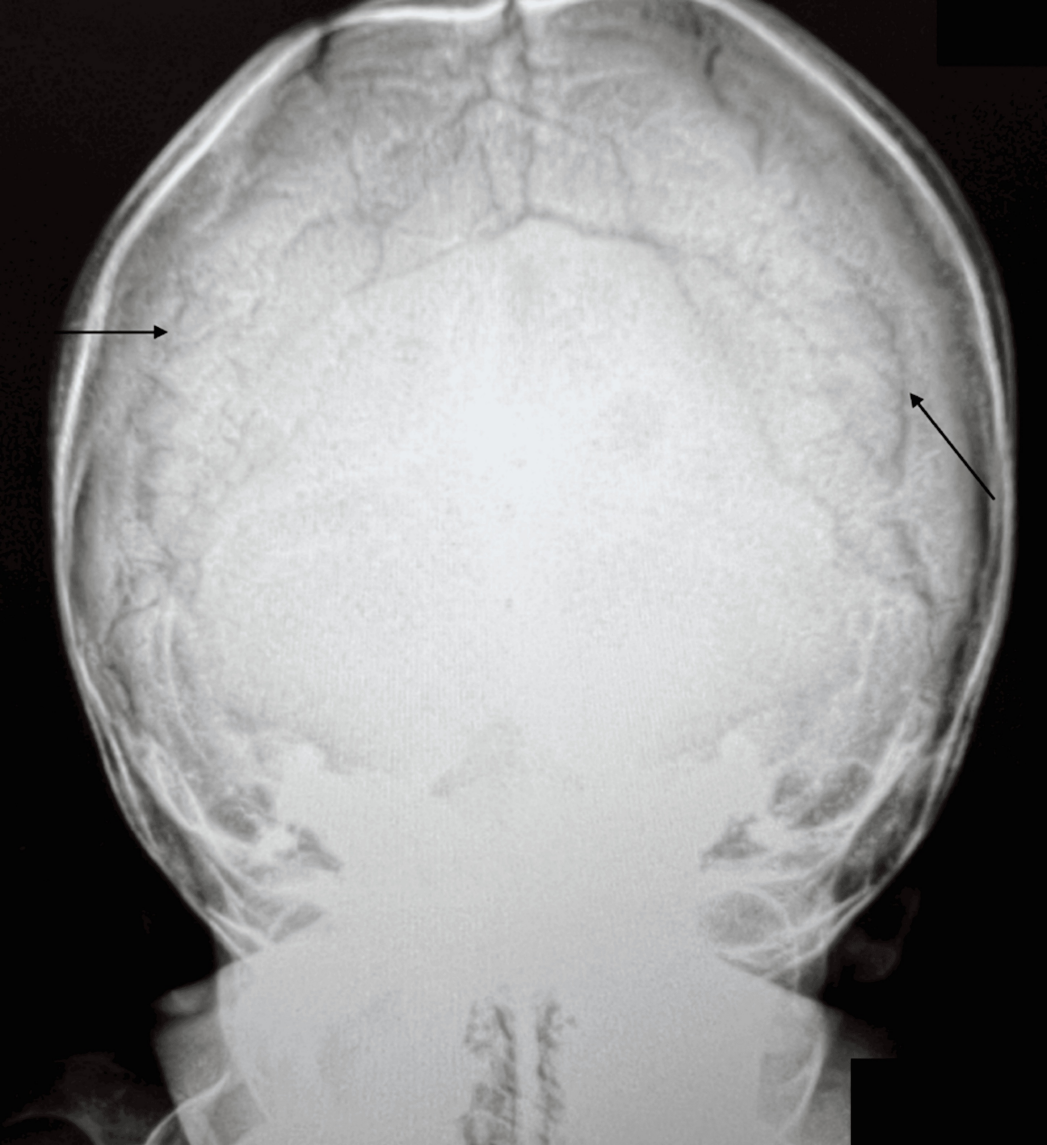
Skull X-ray. Arrows indicate multiple lambdoid Wormian bones.

Subsequent deep phenotyping included skull X-rays, which showed multiple lambdoid Wormian bones (Figure 3). The dual-energy X-ray bone density (Hologic Discovery-W (S/N 83764) Software version: 13.5.3) study revealed bone mineral density measurements slightly above and significantly below -2 SD for age- and gender-matched cohorts for left/right proximal hip and lumbar spine, respectively. Calcium, phosphorus, vitamin D, and alkaline phosphatase levels were all normal at this time.

Following the results at 18 months of age, a one-month trial of celecoxib (50 mg twice daily) failed to relieve symptoms of joint pain and swelling. One dose of zoledronic acid (0.7 mg, administered as a one-time infusion over 30 minutes) was also administered, with no signs of symptom relief. Our patient is currently taking ibuprofen (10 mg/kg per dose every eight hours) but is experiencing suboptimal symptom relief.

## Discussion

The reasons for preferential bone involvement in this syndrome are not fully elucidated. What is known is that PGE2 was found to promote the functions of both osteoclasts and osteoblasts [4]. Furthermore, intrinsic prostaglandin production by osteoblasts activates EP4 receptors, which eventually help secrete norepinephrine [4]. Both these effects seem to increase bone turnover.

Cyclooxygenase-2 (prostaglandin-endoperoxidase synthase 2/COX2) is an inducible enzyme that generates prostaglandins in times of inflammation [7]. COX2 inhibitors were proposed as a treatment for the symptoms of PHOAR1 due to their ability to block prostaglandin production [7]. While in some PHOAR 1/2 patients there was initial remission of some symptoms with COX2-inhibitor treatment, periostitis remained [4,8]. Any lapse of medication usage resulted in the return of symptoms, which, due to side effects of COX2 inhibitors like gastrointestinal upset, make treatment with this class of medication not efficacious long term [8].

Other nonspecific COX inhibitors like ibuprofen, diclofenac, and indomethacin have also been used in patients with PHOAR 1/2 [9]. A systematic review of the use of this class of drug in PHOAR1/2 showed an improvement in arthralgia in about 70% of the patients [9]. However, none of the studies reported any change in hyperhidrosis symptoms [9].

Bisphosphonates have been known to inhibit vascular endothelial growth factors, have anticancer properties, and have been used in the treatment of PHOAR1 [10]. Studies show a gradual, partial response to bisphosphonates in PHOAR1, with some symptom remission of arthritis, swelling, skin thickening, clubbing, and overall pain beginning around three to four days post-treatment and increasing gradually over months to a year [10]. Platelet-derived growth factors seem to induce PGE2 release, which can then stimulate vascular endothelial growth factors, leading to some of the skin changes seen in PHOAR1 [10]. Overall, the treatments for PHOAR 1/2 are suboptimal, and further research into the disease mechanisms and more efficacious treatments are needed.

## Conclusions

Despite being read as a variant of unknown significance, the genotype-phenotype correlation for our patient's *HPGD* variant is identical to that of other patients described with PHOAR1, including his clinical course prior to and subsequent to his genetic diagnosis. This argues for the addition of loss-of-function variants in *HPGD* as a cause of PHOAR1. The rare combination of digital clubbing, PDA, pachydermia, and other arthropathies should prompt both genetic testing and deep phenotyping in search of this unifying diagnosis. Further investigation is needed to find better treatments for this debilitating disease.
